# COVID-19 Parental Vaccine Hesitancy: The Role of Trust in Science and Conspiracy Beliefs

**DOI:** 10.3390/ijerph21111471

**Published:** 2024-11-05

**Authors:** Ambra Gentile, Marianna Alesi

**Affiliations:** Department of Psychology, Educational Sciences and Human Movement, University of Palermo, Viale delle Scienze, ed. 15, 90128 Palermo, Italy; marianna.alesi@unipa.it

**Keywords:** vaccine acceptance, conspiracism, trust in the scientific community

## Abstract

Background. Parent vaccine hesitancy is a sensitive topic despite the benefits associated with children’s vaccination. Especially regarding the COVID-19 vaccination, parents displayed concerns about children’s vaccination, questioning their effectiveness and security. Although several studies were conducted on the general population, few studies investigated this relationship on parents’ intentions. Methods. An online survey was advertised from May to December 2022 on social networks, collecting data from 109 participants (90% F; mean age: 41.34 years, SD: ±6.40). The survey assessed sociodemographic characteristics, vaccine hesitancy through the Parents Attitude towards Childhood Vaccine—PAVC, trust in science through the Belief in Science Scale—BISS, and conspiracy beliefs through the Generic Conspiracist Beliefs Scale—GCBS. Results. In our sample, 29 parents (26.6%) scored more than 50 points to PAVC and, for this reason, were considered hesitant. Moreover, more than half of parents (60.6%) declared that they did not intend to vaccinate their children in the future. The path analysis model showed that parents with low education tended to have higher conspiracy beliefs (β = −0.40). Holding conspiracy beliefs (β = 0.28) and having low trust in science (β = −0.23) was associated with higher parent hesitancy and, in turn, no future intention to vaccinate their children for COVID-19 (OR = 0.83, *p* < 0.001). Conclusion. The results of the current paper suggest that targeted campaigns should be aimed at parents with lower levels of education, mainly on social media, debunking the most common fake news or myths, independently from the type of vaccine, and highlighting the importance of scientific research for improving people’s living conditions.

## 1. Introduction

The COVID-19 pandemic has imposed some practical measures to contrast the spread of the virus, where adult and child vaccination played a primary role [1]. Indeed, the relevance of children’s vaccination regarded the reduced risk of personal contagion [2], as well as the containment of major outbreaks in schools [3] or reduced education disruption (e.g., for limiting difficulties experienced by both teachers and students with distance learning) [4,5]. Regarding the COVID-19 vaccination in the Italian context, the percentage of vaccinated children between the ages of 5 and 11 is around 35%, with 61% of children having never been vaccinated [6].

In general, although vaccines are helpful in fighting serious diseases and preventing deaths, parents might have some concerns about vaccinating their children, which could lead them to become hesitant towards children’s vaccination. Specifically, parental hesitancy, defined as parents’ delay in accepting or refusing children’s vaccination despite the availability of the vaccine [7], is a strong predictor of future intention to vaccinate children [8,9]. According to the studies about parental hesitancy toward vaccination, the most reported concerns for children’s vaccination are related to perceived vaccine risk, including long-term severe side effects [10,11,12]. For instance, Temsah, et al. [13] collected parental worries about COVID-19 vaccination and found that low safety information (69%) and fear of side effects (60.6%) were the most common reasons for hesitancy. In addition, the authors compared parents’ attitudes towards COVID-19 vaccination with their attitudes towards routine vaccination and found that more positive attitudes were held towards routine childhood vaccination than COVID-19 vaccination. Similarly, in a review by Roy, et al. [14], the most commonly reported barrier to COVID-19 vaccination was issues with vaccine safety, followed by doubts about vaccine efficacy, trust in the COVID-19 vaccine, and holding conspiracy beliefs. Other studies highlight the lack of paediatrician recommendations as a crucial determinant of parents’ hesitancy. For example Napolitano, D’Alessandro, and Angelillo [15], concerning the Italian context, reported that hesitancy is associated with a lack of recommendations from a paediatrician, having received conflicting opinions about vaccination, discussing with parents of children experiencing severe side effects, and relying on non-traditional medical treatments. Another Italian study of children aged between 16–36 months found that the main barriers reported by hesitant parents were no recommendations from a paediatrician to fully vaccinate their child, talking with parents of children who experienced vaccine reactions, having received contrasting opinions about vaccination, and accepting non-traditional medical treatments [16].

Regarding the psychological determinants of parental hesitancy, the scientific literature has identified the tendency to believe in conspiracy theories and trust in the scientific community, which are likely to vary according to education level. In particular, Tomljenovic, Bubic, and Erceg [17] observed that holding a lower education level and holding conspiracy beliefs were correlated to stronger unpleasant emotions about vaccination, which were in turn related to vaccine refusal. Indeed, according to the authors, vaccine hesitancy combined with conspiracy beliefs tends to reduce the complexity of reality, including reducing trust in scientific research or accepting alternative research explanations.

Concerning the relationship between parental vaccine hesitancy and trust in the scientific community, Cadeddu, et al. [18] reported that Italians with higher education, who trusted the scientific community, were more likely to disagree about the harmfulness of vaccines. Furthermore, holding a higher degree decreases the probability of believing fake news.

Concerning trust in the scientific community, Galanis, et al. [19] reported that having a higher education level and trusting health science/physicians positively influenced the intention to vaccinate children against COVID-19. Moreover, people with higher education have access to scientific studies and tend to trust the scientific results more than less-educated people.

Although vaccine hesitancy has been extensively discussed in the scientific literature, few studies have been retrieved on the potential psychological process influencing parental vaccine hesitancy in parents. Moreover, considering that the topic is well-known within the scientific literature, further research on parent hesitancy (not only referring to the COVID-19 vaccine) is crucial for maintaining immunity towards the main viruses. Therefore, the current study aims to propose a model of the influence of trust in science and conspiracy beliefs on parental vaccine hesitancy and intention to vaccinate their children. Specifically, we hypothesise that high conspiracy beliefs and low trust in science, depending on parents’ educational level, would correlate with parental hesitancy, which in turn would be negatively associated with parents’ future intention to vaccinate their children.

## 2. Materials and Methods

### 2.1. Participants

Data were collected between May and December 2022 on an initial sample of 132 Italian parents, mainly from Southern Italy. The recruitment was conducted through social networks by asking people with at least one child to answer an online survey. In the case of families with more than one child, parents were required to choose one of them. The inclusion criteria were to be parents and to have children aged between 5 and 15 years. This study was approved by the Bioethical Committee of the University of Palermo (approval nr. 104/2022) and respected the Declaration of Helsinki’s principles.

### 2.2. Measures

#### 2.2.1. Sociodemographic Questionnaire

Sociodemographic information was detected through an ad-hoc questionnaire created by the Authors. The items collected information about age, civil status, education, annual income, number of children, age of children, and number of shots received. The intention to vaccinate children was assessed through a dichotomic question (“Do you intend to vaccinate your child against COVID-19 in the future?”).

#### 2.2.2. Parents Attitude Toward Childhood Vaccine (PAVC)

The Parents Attitude towards Childhood Vaccine—PAVC [20] is a 15-item self-report scale, with hesitant responses scored 2, ‘don’t know or not sure’ scored 1, and non-hesitant responses scored 0 (e.g., “Have you ever delayed having your child get a shot for reasons other than illness or allergy?”). Total scores were normalized on a scale from 0 to 100 with simple linear transformation, where a score ≥ 50 identified hesitant parents and a score < 50 indicated non-hesitant parents. The scale has a good internal consistency (α = 0.85).

#### 2.2.3. Generic Conspiracist Beliefs Scale

The Generic Conspiracist Beliefs Scale—GCBS [21] a 15 self-reported items measured on a 5-point Likert scale (from 1 “definitely not true” to 5 “definitely true”). A sample item is “The power held by heads of state is second to that of small unknown groups who really control world politics”. Total scores were obtained by raw sum, with scores ranging from 15 to 75. The scale showed an excellent internal consistency (α = 0.92).

#### 2.2.4. Belief in Science Scale

The Belief in Science Scale—BISS [22] was used to assess parents’ trust in science. The scale consists of 10 unidimensional items assessed on a 6-point Likert scale (from 1 “strongly disagree” to 6 “strongly agree”; e.g., “We can only rationally believe in what is scientifically provable”). Total scores are obtained through the raw sum, ranging from 10 to 60. Higher scores indicate a stronger belief in science. The scale has an excellent internal consistency (α = 0.92).

### 2.3. Data Analysis

Data were analysed using SPSS (version 24, IBM, New York, NY, USA). Descriptive statistics were obtained by calculating frequencies on the main sociodemographic variables. Chi-squared test and *t*-test were used to detect significant differences between the hesitant and non-hesitant groups. Correlations between variables were calculated using Pearson correlation. A path analysis model was run with MPlus—version 7 [23], hypothesising that high conspiracy beliefs and low trust in science, both linked to parents’ educational level, would be correlated with parental hesitancy, which in turn would be negatively associated with parents’ future intention to vaccinate their children.

## 3. Results

### 3.1. Descriptive Statistics

From descriptive statistics, 23 parents were excluded for not respecting the inclusion criteria, resulting in a final total sample of 109 participants. The respondents were mainly female (90%), with a mean age of 41.34 years (±6.40), and married (90%). Participants reported having a high school diploma (47%) or university degree (36%), and an annual income between 20,000 and 40,000 euros. The respondents declared having 2 children (60%), aged between 5 and 15 years (mean age: 8.84 ± 2.99) (Table 1). From the initial analysis, 29 parents (26.6%) had a PAVC score > 50, therefore they were identified as “hesitant”, while the other 80 parents (73.4%) were categorised as “non-hesitant”.

### 3.2. Health Status

Concerning the health status, 75 parents (68.8%) declared having been sick with COVID-19, 18 of whom were hesitant. Moreover, almost all parents were vaccinated for COVID-19 (94.5%), both hesitant and non-hesitant (see Table 2).

Regarding their children, 62.4% of parents declared that their children had COVID-19, 18 of whom were hesitant, and 47 parents declared that they did not vaccinate their children. There is a significant association between children’s past vaccination against COVID-19 and being hesitant/non-hesitant (Chi-squared = 3.87, *p* < 0.05).

### 3.3. Intention, Conspiracy Beliefs, and Trust in Science

More than half of the respondents (60.6%) declared having no intentions of vaccinating their children in the future, from both hesitant and non-hesitant parents. Significant differences were found between hesitant and non-hesitant parents in terms of conspiracy beliefs and trust in science. Specifically, hesitant parents held conspiracy beliefs to a greater extent (*t* = −2.85, *p* < 0.01) and had less trust in science (*t* = 10.33, *p* < 0.001) than non-hesitant parents (see Table 3).

Table 4 shows the correlation between parents’ hesitancy scores, conspiracy beliefs, trust in science, and education level. Hesitancy scores were significantly and positively related to conspiracy beliefs (r = 0.38, *p* < 0.001) and negatively related to trust in science (r = −0.23, *p* = 0.015) and education level (r = −0.27, *p* = 0.004). Conspiracy beliefs were negatively related to education level (r = −0.40, *p* < 0.001), while trust in science was not related to education level.

### 3.4. Path Analysis Model

**Figure 1:**
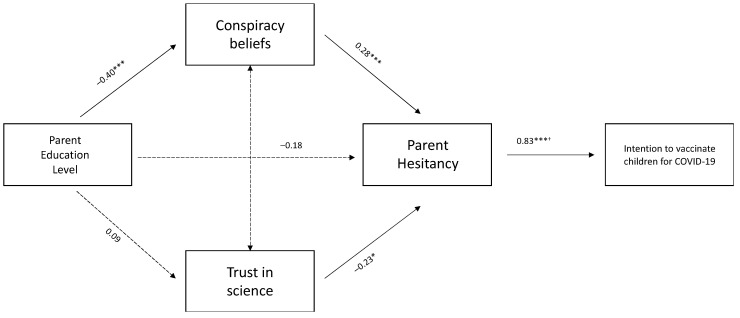
Path analysis model for the intention to vaccinate children against COVID-19. Legend: *** *p* < 0.001; * *p* < 0.05; all the values are betas, except for ^†^ which is odds ratio.

A path analysis model (Figure 1) was run for the influence of education level, conspiracy beliefs, trust in science, and hesitancy on the intention to vaccinate children in the future. The model showed an excellent fit (χ^2^ = 82.41, *p* < 0.001; CFI = 0.99, TLI = 0.98, RMSEA = 0.03). Specifically, the education level is negatively associated with conspiracy beliefs (β = 0.40, *p* = 0.001), but was not associated with trust in science (β = −0.09, ns), nor parental hesitancy (β = −0.18, ns). Conspiracy thinking was positively related to parental hesitancy (β = 0.28, *p* = 0.001). Similarly, trust in science was negatively related to parental hesitancy (β = −0.23, *p* = 0.03), which in turn was significantly associated with the intention to vaccinate children in the future against COVID-19 (OR = 0.83, *p* = 0.001). In other words, parents with low education and high conspiracy beliefs or with low trust in science tended to be hesitant and had no intention to vaccinate their children for COVID-19 in the future.

## 4. Discussion

The current study investigated some potential psychological processes underlying parental vaccine hesitancy. In particular, we hypothesised that high conspiracy beliefs and low trust in science, both linked to parents’ educational level, would be correlated with parental hesitancy, which in turn would be negatively associated with parents’ future intention to vaccinate their children. First, in our sample, 26.6% of the respondents were categorized as hesitant, which is similar to the result reported in the scientific literature. For example, Bianco, et al. [24] reported that 12.4% of the interviewed Italian parents were hesitant, while Montalti, et al. [25] reported a percentage of 9.9% of Italian hesitant parents in their survey. Moreover, more than half of the respondents (60.6%) declared to have no intention of vaccinating their children against COVID-19. Indeed, vaccination against COVID-19 raised many concerns mainly due to the small time employed for testing its efficacy [26]. For example, Temsah, et al. [13] compared the acceptance rate of routine vaccines vs. COVID-19 vaccination, finding a higher acceptance of routine vaccines, and higher concerns and anxiety about COVID-19 vaccine side effects compared to those of routine vaccines.

Concerning the path analysis model, it seems that holding a lower education degree and high conspiracy beliefs, together with low trust in science, might increase parental hesitancy, which, in turn, can influence the intention to vaccinate children. Similarly to our results, Santirocchi, et al. [27] reported a significant correlation of trust in science and conspiracy beliefs with vaccine hesitancy and intention. Moreover, the role of conspiracy beliefs and trust in science was confirmed in the Serbian context by Đorđević, et al. [28], finding that vaccine hesitancy about any vaccine-preventable disease was directly influenced by high conspiracy beliefs and low trust in science both for adults and for children vaccination. Concerning conspiracy beliefs, the study of Tomljenovic, Bubic, and Erceg [17] highlighted that conspiracy beliefs seem to be connected to unpleasant emotions towards vaccination, greater experientially intuitive thinking, combined with lower levels of education. This phenomenon might have been increased by mass media and social media by spreading rumours, myths, and inaccurate beliefs about vaccines [29]. Indeed, Kouzy, et al. [30] collected around 673 tweets about COVID-19 and estimated that more than 24% contained mistakes in spreading information. Other conspiracy beliefs associated with COVID-19 vaccine and spread through social media related to the involvement of 5G technology within the vaccine and the pandemic as a plan by pharmaceutical companies aiming to sell their vaccines [31]. Therefore, misinformation, together with the tendency to believe in conspiracism, might have undermined COVID-19 vaccine acceptance.

Regarding trust in science, similar results were reported by Carrieri, Guthmuller, and Wübker [32], who found a negative correlation between trust in science and vaccine hesitancy. On the contrary, Cadeddu, Sapienza, Castagna, Regazzi, Paladini, Ricciardi, and Rosano [18] found that a higher vaccination rate was linked to higher trust in the scientific community. Taking these results together, trust in science, which is likely to be lower in people with lower degrees, is connected to vaccine hesitancy or refusal.

We did not find any relation between parents’ education level and trust in science, both in Pearson’s r correlation and in the path analysis model. Other studies found weak or no relation between education level and trust in science [33,34]. According to van Mulukom, et al. [35], this relation might depend on the social context. In the case of Italy, the pandemic course, characterized by multiple lockdowns and strict restrictions (e.g., indoor and outdoor mandatory face masks), might have determined questions about the effectiveness of the pandemic measures proposed by the government and the scientific community. In this way, trust in the scientific community could have been temporarily low across all the education levels.

### Limitations

As previously mentioned, this is the first study that hypothesises a model that highlights the processes of parents’ hesitancy. However, this study has some limitations. First of all, this study employs a small sample size, resulting from a brief recruitment phase given that the study was conducted at the very end of the pandemic, when the contagion rate started decreasing. Moreover, the sample size was unbalanced in terms of the gender of the respondents who were mainly females. Previous studies highlighted that females tend to participate in surveys [36] and in particular health research surveys when recruited through Facebook [37]. Moreover, according to previous studies, mothers tend to be more hesitant than fathers [25,38], therefore a more balanced sample could offer more cautious results. Secondly, we did not assess if children received the routine vaccination. This information would have been useful for understanding if the hesitancy toward the COVID-19 vaccine would overlap with the hesitancy towards general vaccination, similar to Temsah [13], which is the only study comparing attitudes towards the COVID-19 vaccine with routine vaccines.

Finally, we did not control for other variables, such as fear of COVID-19 or anxiety, that could have increased vaccine acceptance [39].

## 5. Conclusions

The current paper highlighted the crucial role of trust in science and conspiracy beliefs in influencing parental hesitancy, which, in turn, seems to be related to the intention of vaccinating children in the future.

To increase vaccine acceptance among parents, a targeted vaccination campaign should be aimed at parents with lower levels of education, primarily through social media and television, debunking the most common fake news or myths. In parallel, the campaign should highlight the benefits that COVID-19 vaccination has had on global living conditions and the improvements introduced by the scientific community.

## Figures and Tables

**Table 1:** Sociodemographic characteristics of the sample.

	Total Sample (*n* = 109)	Hesitant (*n* = 29)	Non-Hesitant (*n* = 80)	Chi-Squared Test/ *t*-Test	*p*-Value
Gender				0.03	0.95
*Males*	11 (10.1%)	3 (10.3%)	8 (10.0%)		
*Females*	98 (89.9%)	26 (89.7%)	72 (90.0%)		
Age	41.34 ± 6.40	38.24 ± 6.65	42.46 ± 5.96		
Education level				4.00	0.26
*Middle school diploma*	8 (7.3%)	4 (13.7%)	4 (5.0%)		
*High school diploma*	51 (46.8%)	14 (48.3%)	37 (46.2%)		
*University degree*	39 (35.8%)	10 (34.5%)	29 (36.2%)		
*Post-lauream title*	11 (10.9%)	1 (3.5%)	10 (12.6%)		
Annual family income (in euros)				7.52	0.06
*0–20,000*	30 (27.5%)	10 (34.4%)	20 (25.0%)		
*21,000–40,000*	52 (47.7%)	17 (58.6%)	35 (43.8%)		
*41,000–50,000*	23 (21.1%)	1 (3.5%)	22 (27.4%)		
*More than 50,000*	4 (3.7%)	1 (3.5%)	3 (3.8%)		
Marital status				8.62	0.04 *
*Married*	98 (89.9%)	23 (79.3%)	75 (93.6%)		
*Divorced*	6 (5.5%)	2 (6.9%)	4 (4.6%)		
*Common-law partner*	3 (2.8%)	2 (6.9%)	1 (0.9%)		
*Single*	2 (1.8%)	2 (6.9%)	0 (0.0%)		
Number of children				1.43	0.49
*1*	34 (31.2%)	11 (39.3%)	23 (29.8%)		
*2*	63 (57.8%)	16 (57.1%)	47 (61.2%)		
*3*	8 (7.3%)	1 (3.6%)	7 (9.0%)		
Mean age of the reference child	8.84 ± 2.99	8.39 ± 2.91	9.00 ± 3.02		

Chi-squared significant differences at; * *p* < 0.05.

**Table 2:** Health status of the sample.

	Total Sample (*n* = 109)	Hesitant (*n* = 29)	Non-Hesitant (*n* = 80)	Chi-Squared Test/*t*-Test	*p*-Value
Have you ever got sick with COVID-19?				0.83	0.36
*Yes*	75 (68.8%)	18 (62.0%)	57 (70.0%)		
*No*	34 (31.2%)	11 (38.0%)	23 (30.0%)		
Has your child ever got sick with COVID-19?				0.02	0.97
*Yes*	68 (62.4%)	18 (62.0%)	50 (62.5%)		
*No*	41 (37.6%)	11 (38.0%)	30 (37.5%)		
Have you ever been vaccinated against COVID-19?				0.15	0.65
*Yes*	103 (94.5%)	27 (93.1%)	76 (95.0%)		
*No*	6 (5.5%)	2 (6.9%)	4 (5.0%)		
Has your child ever been vaccinated against COVID-19?				3.87	0.05 *
*Yes*	62 (56.9%)	12 (41.3%)	50 (62.5%)		
*No*	47 (43.1%)	17 (58.4%)	30 (37.5%)		

* Chi-squared significant difference at *p* < 0.05.

**Table 3:** Intention, conspiracy beliefs, and trust in science scores.

	Total Sample (*n* = 109)	Hesitant (*n* = 29)	Non-Hesitant (*n* = 80)	Chi-Squared Test/*t*-Test	*p*-Value
Do you intend to vaccinate your child against COVID-19 in the future?				14.01	<0.001 ***
*Yes*	43 (39.4%)	3 (10.3%)	40 (50.0%)		
*No*	66 (60.6%)	26 (89.7%)	40 (50.0%)		
Conspiracy beliefs **	25.82 ± 10.03	29.48 ± 6.93	24.49 ± 10.66	−2.85	0.006 **
Trust in science *	39.43 ± 11.37	35.45 ± 11.33	40.88 ± 11.10	2.24	0.03 *

*** *p* < 0.001; ** *p* < 0.01; * *p* < 0.05.

**Table 4:** Correlations among variables.

	Hesitancy	Conspiracy Beliefs	Trust in science	Education Level
1- Hesitancy	-	0.38 ***	−0.23 *	−0.27 **
2- Conspiracy Beliefs		-	−0.12	−0.40 ***
3- Trust in science			-	0.09
4- Education level				-

Legend: *** *p* < 0.001; ** *p* < 0.01; * *p* < 0.05.

## Data Availability

Data are available upon formal request addressed to segreteria.comitato.bioetica@unipa.it.
